# Notes on the Treatment of Diphtheria

**Published:** 1906-03-03

**Authors:** 


### Hospital Clinics.

### NOTES ON THE TREATMENT OF DIPHTHERIA.

In a paper on this subject Dr. T. Basil Rhodes gives some sound practical advice based on his own personal experience. He urges the necessity for early administration of antitoxin in a sufficiently large dose in every suspected case. With definite membrane on the tonsil, or with profuse and thick nasal discharge, cases are at once recognised and treated. But in less obvious cases it is often the practice to wait for the result of a bacteriological examination before using antitoxin, and in this way valuable time is lost. It is the early use of the remedy which is of prime moment. Given at the outset in a "suspected" case the symptoms will be favourably modified, or will even disappear, whereas if the case is allowed to become "obvious" the patient may be in such a condition of toxæmia that antitoxin even in large doses is too late. It may be taken as certain that in any ordinary case of faucial diphtheria if antitoxin (2,000 units) be administered on the first day of the disease, or 4,000 units on the second day, there is little or no danger for the child, provided that rest and other suitable methods of after-treatment are practised. If antitoxin—4,000 or 6,000 units—is given on the third day the case will probably proceed to a favourable recovery, though a second dose may be found necessary. On the fourth day, as a rule, 8,000 units may be necessary. On the fifth day it is well to give 6,000 or 8,000 units, and to repeat this dose six or eight hours later. On the sixth day toxæmia is usually so advanced that antitoxin will hardly overtake it. In the laryngeal form of the disease, especially when the infection has spread from the nostrils or naso-pharynx, even greater urgency exists; sometimes a matter of two days will place such a case on the hopeless list. To reinforce this advice it may be said that antitoxin never does any harm. Abscess formation at the site of injection can be avoided by cleanliness and care; urticarial and other rashes and joint pains are infrequent, and have no permanent ill-results; and the statement that antitoxin causes paralysis is contrary to fact.

The other measures advisable in the treatment of diphtheria may be summarised as follows:—The throat, and, if there is nasal discharge, also the nose, may be frequently douched with boric acid solution; in addition, the throat may be occasionally swabbed with a solution of sodium bicarbonate (a teaspoonful to a pint of water). But in connection with these suggestions it must be remembered that to upset the patient is above all things bad policy, and hence they may have to be suspended or modified when they cause much disturbance. Rest is of the highest importance, and raising the foot of the bed both quiets the patient and assists the action of the heart. Even with mild attacks great caution must be exercised. A good rule is to keep the child lying in bed without a pillow for at least a fortnight, then allow one pillow in the third week, a second in the fourth week, and a third after a further five or six days. Then the patient may be moved on to a couch, and after a few days, if all goes well, may be permitted to walk a little. In some instances the recumbent position must be maintained for a much longer period. A soft pulse, or one that readily quickens, is a ground for caution, and attention must also be paid to the patient's general condition. In regard to internal medication purgatives are best avoided after the first few days, as they sometimes bring on vomiting which may be difficult to control; enemata are to be preferred. Iron is best avoided in the early stages, but is useful at a later date.

*The treatment of sequelæ and complications.*—Diphtheritic paralysis calls for rest of the whole body, and, so far as possible, for rest of the involved muscles, and for the administration of strychnine. In paralysis of the soft palate with regurgitation of liquid foods a jelly made of milk and isinglass, sweetened, and if necessary, with brandy added is easily swallowed. Nasal feeding is much resented, and is rarely necessary. With signs of rapidly failing heart—often attended with uncontrollable vomiting—strychnine (1-30th grain or more) hypodermically is most valuable, as is also brandy. The latter may be given with peptonised milk as an enema, or where this cannot be retained it may be injected (20 minims) under the skin alternately with the strychnine. The "rheumatism" or arthritis which sometimes follows diphtheria is probably the result of antitoxin; it is best treated by such local measures as lead and opium lotion. In laryngeal diphtheria hot fomentations over the larynx, steam inhalations with compound tincture of benzoin, and stimulants will often carry a child through until the antitoxin has time to come into operation, and will thus enable tracheotomy to be avoided.

[1] Brit. Med. Jour., Feb. 17, 1906.

